# Design of DOX-GNRs-PNIPAM@PEG-PLA Micelle With Temperature and Light Dual-Function for Potent Melanoma Therapy

**DOI:** 10.3389/fchem.2020.599740

**Published:** 2021-01-05

**Authors:** Na Wang, Jing Shi, Cong Wu, Weiwei Chu, Wanru Tao, Wei Li, Xiaohai Yuan

**Affiliations:** ^1^Department of Cosmetics, Shanghai Skin Disease Hospital, Shanghai, China; ^2^Laboratory of Nano Biomedicine & Intentional Joint Cancer Institute, Second Military Medical University, Shanghai, China

**Keywords:** doxorubicin, gold nanorods, thermosensitive, photothermal therapy, melanoma, B16F10

## Abstract

**Objective:** The aim of this study was to construct light and temperature dual-sensitive micellar carriers loaded with doxorubicin (DOX) and gold nanorods (DOX-GNRs-PNIPAM@PEG-PLA, DAPP) for melanoma therapy.

**Methods:** The DAPP self-assembled using fine-tuned physicochemical properties in water. The DAPP structure, temperature- and photo-sensitivity, drug-release, *in-vitro* serum stability, and cytotoxicity against melanoma B16F10 cells were evaluated in detail. The corresponding *in-vitro* and *in-vivo* therapeutic mechanisms were then evaluated using a B16F10-melanoma bearing BALB/c nude mouse model (B16F10).

**Results:** The light and temperature sensitive micellar drug-delivery system assembled from block copolymer and gold nanorods exhibited a narrow particle size and size distribution, good biocompatibility, well-designed photo-temperature conversion, controlled drug release, and high serum stability. Compared with the free DOX- and PBS-treated groups, the cell endocytosis-mediated cytotoxicity and intra-tumor accumulation of DAPP was markedly enhanced by the NIR-light exposure and induced potent *in-vivo* tumor inhibitory activity.

**Conclusion:** The design of DAPP, a dual-functional micellar drug-delivery system with temperature- and light-sensitive properties, offers a new strategy for skin-cancer therapy with a potent therapeutic index.

## Introduction

Skin cancer is one of the most common aggressive malignancies with an insidious and high morbidity (Siegel et al., 2019). Early melanoma is largely curable with surgery. However, most patients are in the advanced stages when they are diagnosed and lesions are difficult to fully excise due to a series of complications (Yang et al., 2016; Smith et al., 2018). The current treatment approach includes surgery, radiotherapy, chemotherapy, and immunotherapy (Ma et al., 2018; Pinho et al., 2019). Conventional chemotherapy drugs, especially doxorubicin (DOX), are widely used in clinical practice and have good therapeutic efficiency. However, the poor bioavailability and poor tumor targeting of DOX produces a series of side effects, such as nausea, vomiting, bone marrow suppression, and cardiac toxicity, which limits its clinical merit in the treatment of melanoma. Therefore, it is an urgent unmet need to find novel strategies to reduce the clinical limitations of DOX treatment of melanoma.

Nano-based formulations have offered new hope to overcome the abovementioned shortcomings in DOX treatment of melanoma through strategies that involve the protection of therapeutic agents from degradation, improvement of drug bioavailability, and prolonged intratumoral retention (Hou et al., 2015; Pautu et al., 2017; Zhang et al., 2019b). Many nanoformulations have been developed recently for melanoma therapy. Chen et al. synthesized pH-responsive micelles to improve DOX-release when stimulated by the acidic tumor microenvironment of melanoma. The micelles proved to be stable at blood pH (~7.4) and drug release was promoted at an acidic pH (Chen et al., 2013). A new immune gold nanoparticle (AuNP) with photodynamic therapeutic potential was generated against melanoma B16F10 cells, which induced hyperthermal therapy and stimulated an anti-tumor immune response (Zhang et al., 2019a). Other functional and targeted nanodrug delivery systems have also been extensively developed recently against solid melanoma and metastases with a potent therapeutic index and clinical applicability (Long et al., 2018). All these newly developed nanomedicines have illustrated the exciting potential for treatment of melanoma.

Conversely, Chan et al. reported that the intertumoral accumulation of nanotherapeutics was equal or lower than 0.7%, which differed from that expected from previously reported studies (Wilhelm et al., 2016). Subsequently, different nano-based formulations have been designed to improve the intratumoral accumulation of drugs, promote *in-vivo* bioavailability, enhance circulation time and drug release at the tumor site. The unique composition and properties of the melanoma tumor microenvironment (TME) is characterized by hypoxia, acidosis, and high temperature (Li et al., 2011b, 2012b; Deng et al., 2017). The TME plays a major role in the occurrence, invasion, and metastasis of tumors and has provided a rationale for in the development of multifunctional nanoparticles (Li et al., 2012a,c; Yang and Gao, 2017). An interesting study by Guangjun et al. designed a nanoparticle carrying the anti-platelet antibody R300 and chemotherapeutic drug DOX. The formulation could locally deplete tumor-related platelets, which consequently enhanced vascular permeability with a high enhanced permeability and retention (EPR) effect and drug accumulation at the tumor sites (Li et al., 2017). In contrast, the strategies of *in-situ* destruction of tumor tissue and enhancing the EPR may also lead to damage to normal cells and tissues with their associated undesired side effects. This indicates the importance of promoting intertumoral drug release without any *in-vivo* blood or non-tumor tissue damage through physical stimulation, such as using gold nanorods (GNRs) with a strong photothermal conversion effect (Zhang et al., 2016, 2017). In our previous studies, we found that the intratumoral drug accumulation was enhanced by temperature-sensitive passive targeting (TSPT) micelles composed of thermosensitive poly(N-isopropylacrylamide) (PNIPAM)(Li et al., 2011a, 2012d). Tumor accumulation can be enhanced further by the fine-tuning of the carrier based on the “Li-Teruo” plot (Li et al., 2011a,b). This indicates the potential for potent tumor accumulation of drugs by combining the photothermal conversion of GNRs with micelles having a PNIPAM-based core.

Herein, a dual-functional micellar drug delivery system based on PNIPAM, poly (d, l-lactide)-poly (ethylene glycol) (PLA-PEG), and GNRs was constructed by incorporating of DOX, GNRs, and PNIPAM into the core of PEG-PLA micelles (DAPP), equipped with temperature, and light dual-sensitive properties. The heat generated by the GNRs in the DAPP induces shrinking of the PNIPAM, which in turn will promote drug release and achievement of higher local drug concentration at the tumor site controlled by *in-situ* near-infrared (NIR)-light stimulation. The thermo-enhanced synergistic treatment against B16F10 cells by GNRs and DOX will result in no damage to off-target normal cells.

## Materials and Methods

### Main Chemicals and Apparatus

Doxorubicin hydrochloride (DOX·HCl) was purchased from Dalian Meilun Biotechnology Co., Ltd. Bovine serum albumin (BSA) was purchased from J&K Scientific Ltd. GNRs, PNIPAM, and PEG-PLA were previously synthesized by our colleagues. Human melanoma cells B16F10 were purchased from the American Type Culture Collection. Dulbecco's Modified Eagle's Medium (DMEM) and fetal bovine serum (FBS) were purchased from Gibco Co, United States. The Cell Counting Kit-8 was purchased from Dojindo Laboratories (Kumamoto, Japan). The apparatus mainly consisted of a UV-VIS spectrophotometer (Cary300, Varian, CA, United States), a freeze dryer (VirTis AdVantage, United States), a dynamic light-scattering device (DLS, ALV/CGS-3, Germany), an infrared light source (IRS-S6, Shanghai, China), a transmission electron microscope (TEM) (Hitachi, H-7000 Electron Microscope), an inverted fluorescence microscope (Olympus IX71, Japan), and an enzyme-labeling instrument (Power Wave XS, Bio-TEK, United States).

### Preparation of DAPP

The PNIPAM aqueous solution (2 mL) was mixed with 2.0 mg DOX and 2.0 mg GNRs. After thorough mixing, the DOX and GNRs were stirred for 4 h at room temperature in the dark, so that the DOX and GNRs were fully incorporated with the PNIPAM (Han et al., 2017a; Chen et al., 2020). The mixture was then dried by freeze-drying to form solid powder (Li et al., 2012d). Subsequently, 4 mg/mL PEG-PLA amphiphilic block copolymer solution (DMAC) was mixed with the PNIPAM/DOX/GNRs powder and stirred at room temperature to form the nanoparticles (Gong et al., 2019). After a 6-h reaction time, the solution was dialyzed with a dialysis bag (MW cut off 3500) against phosphate belanced solution (PBS) for 12 h in order to obtain the DAPP solution and to remove any unreacted polymer (Su et al., 2017).

### Characterization of DAPP

GNRs and DAPP were diluted with pure Milli-Q water at a concentration ~0.1 mg/mL. Next, the hydrodynamic diameter and particle size distribution of GNRs, DAPP, and DAPP in the presence of a NIR-light solution was evaluated by DLS at the scattering angle of 90°. The morphology of GNRs and DAPP particles was detected by TEM and the conventional TEM images were obtained at 200 kV with a magnification of 80,000 times. The photothermal conversion efficiency of GNRs and DAPP at different times and concentration of 10, 50, and 100 μg/mL was tested by infrared system at the power of 1,200 mV (Cao M. et al., 2015; Cabral et al., 2018).

### Temperature-Sensitive Properties

The temperature-sensitive properties of PNIPAM and DAPP micelles was measured by DLS with temperature-controller (Li et al., 2011a). The radius of the PNIPAM and DAPP solution (~1 mL, concentration ~0.1 mg/mL) at different temperatures were detected at the scattering angle of 90° (Zhu et al., 2017).

### Serum Stability of DAPP

BSA was used as a protein in the simulated systemic circulation to detect serum stability of DAPP. The solutions of BSA (50 mg/mL) mixed with DAPP micelles (0.1 mg/mL) in PBS were measured by DLS at the scattering angle of 90° at the time of 24 and 48 h.

### *In-vitro* Drug Release

The evaluation of *in-vitro* drug release was conducted using the dialysis method. One set of DAPP was illuminated with NIR-light for 5 min before sampling at certain time intervals while the other received no NIR-light. A volume of 4 mL of DAPP micelles solution was added to the dialysis bag. The dialysis bag was placed into a beaker and was subjected to dialysis with 1,000 mL PBS, with the beaker temperature maintained constant at 37°C in the oil bath with stirring. At the predesigned time 0,1, 2, 4, 6, 12, 24, and 36 h, 100 μL of the micelle solution was removed from the dialysis bag. Simultaneously, about 100 μL PBS was added to the DAPP micelles solution inside membrane and 100 μL acetonitrile was added to the collected micelles solution to break the micelles and release DOX. The solution was then analyzed by UV-VIS spectrophotometer. The DOX release was calculated using the following equation:

(1) $$\begin{array}{r} Drug_{release}=\frac{M_{T}-M_{R}}{M_{T}}\;\times 100\% \end{array}$$

Where *M*_*R*_ is the DOX concentration, and *M*_*T*_ the initial DOX concentration of DAPP micelles.

### Endocytosis

B16F10 cells were seeded into a confocal culture dish (5 × 10^3^cells/dish) with 2.0 mL DMEM medium and cultured for 24 h. Next, the cells were treated with DOX, DAPP, and DAPP with NIR-light and incubated for 2 h at 37°C. The culture medium of B16F10 cells was aspirated, and cells were rinsed with PBS twice, fixed for 10 min with 1.0 mL paraformaldehyde solution, followed by rinsing with PBS three times. The nuclei of the B16F10 cells were stained with DAPI working solution for 10 min, and the B16F10 cells were visualized by inverted fluorescence microscope (Han et al., 2017b).

### *In-vitro* Cytotoxicity

The growth inhibitory effect of DOX, GNRs exposed to NIR-light, and DAPP exposed to NIR-light on B16F10 tumor cells *in vitro* was determined using the CCK-8 assay. B16F10 cells were digested and seeded onto 96-well plates (5 × 10^4^ cells/mL, 0.1 mL/well) and incubated overnight till the cells reached confluence. The samples of DOX, GNRs, and DAPP were diluted by DMEM with concentration of 2, 4, 6, 8, and 10 μg/mL, the medium was removed, and the diluted sample solution of different concentrations were added to the 96-well plates and incubated for an additional 24 h. The GNR and DAPP groups were exposed to 1,200 mV NIR for 5 min, while other groups were without NIR. The medium was incubated with 10% CCK-8 at 37°C for about 0.5–1 h. The optical density (OD) value of 96-well plates was measured at the wavelength of 450 nm by an enzyme-labeling instrument (Kinoh et al., 2020; Mi et al., 2020). The cell viability was calculated using the following equation:

(2) $$\begin{array}{r} Cell_{viability\;}\%=\frac{A_{S}-A_{B}}{A_{C}-A_{B}}\times 100\% \end{array}$$

Where *A*_*S*_, *A*_*B*_, and *A*_*C*_ were the absorption values of the drug treatment groups, culture medium, and cells without drug, respectively.

### *In-vivo* Distribution and Antitumor Effects of DAPP in BALB/c Nude Mice

BALB/c nude mice (4 weeks) were allowed to acclimate for 1 week in the animal facility to reduce stress. The melanoma-bearing murine model was established by subcutaneously implanting 1 × 10^7^ B16F10 cells into the right back (Cao P. et al., 2015; Han et al., 2018). After 10 days, the mice were divided randomly into three treatment groups (three nude mice per group) for *in-vivo* distribution experiment: free fluorescein isothiocyanate (FITC), DAPP, and DAPP with NIR-light, and *in-vivo* anti-tumor effect experiment: PBS, DAPP, and DAPP with NIR-light. As the tumor volume (following equation 3) reached about 100 mm^3^, the mice were injected with FITC and DAPP by tail vein, then the DAPP-treated group was illuminated with NIR-light for 5 min at the laser irradiation of 1,200 mV while the others were not exposed to NIR-light. After 4 h, the mice were anesthetized by isoflurane, and *in-vivo* tumor accumulation was tested by IVIS® Lumina II Imaging System (Xenogen) at an excitation wavelength of 474 nm. For the anti-tumor experiment, mice were injected with PBS and DAPP (0.1 mg/mL) at the dose of 50 mg/kg from the tail vein, and the DAPP group was irradiated by NIR with power of 1,200 mV for 5 min every 2 days. The volume of tumor tissue was measured every 2 days using a digital vernier from the first day of injection to the last day, and the relative tumor volume was calculated according to the following equation:

(3) $$\begin{array}{r} V_{tumor}={Width}^{2}\times Length/2 \end{array}$$

(4) $$\begin{array}{r} V_{relative}=V_{tumor}/V_{initial} \end{array}$$

Where *width* and *length* were the longest and shortest diameters of tumor tissue. V_*initial*_ is the volume of first day injection therapy drug. The tumor progression was evaluated in terms of relative tumor volume (Equation 4), and the body weight of all mice was also recorded to analyze the systemic toxicity of treatment.

All mice were purchased from the Shanghai Experimental Animal Center of Chinese Academic of Sciences (Shanghai, China). The animals were maintained in a pathogen-free environment and allowed to acclimate for at least one week before tumor implantation. The animal study was reviewed and approved by the Institutional Review Board of the Second Military Medical University.

### Statistical Analysis

Statistical analysis was performed by Student's *t*-test or one-way ANOVA to identify significant differences. A *p* < 0.05 was considered statistically significant.

## Results and Discussion

### Preparation and Characterization of DAPP

**Figure 1:**
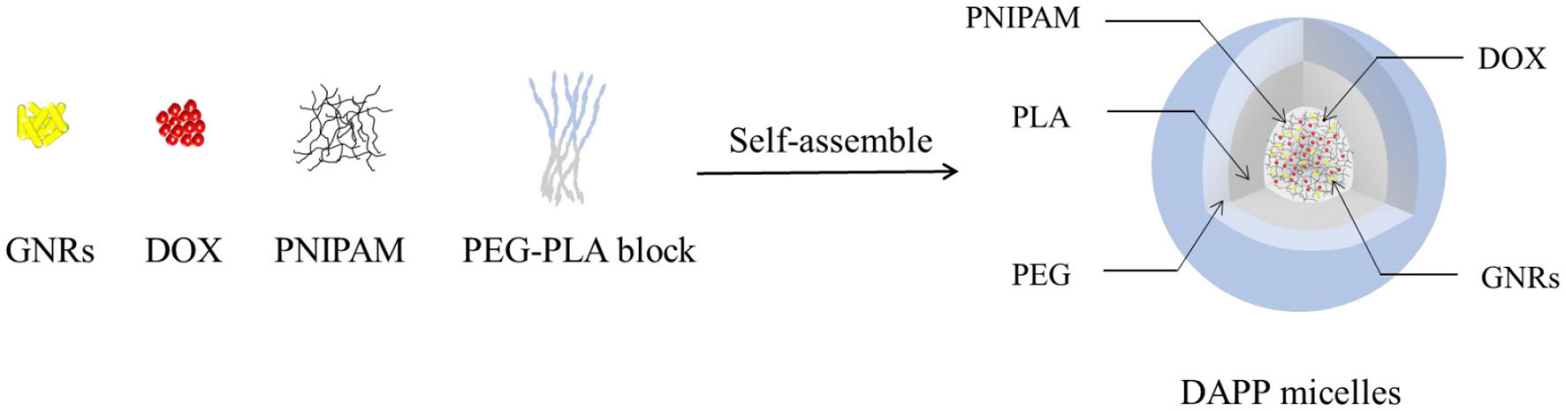
The scheme illustrates the structure of DAPP with temperature and light dual-function.

The structure and composition of DAPP are illustrated in Figure 1. The GNRs and DOX were first mixed with PNIPAM. The mixture was then encapsulated into the core of the PEG-PLA micelles by adding them to the amphiphilic polymer PEG-PLA solution, and then blended and stirred. The physicochemical properties of the DAPP were adjusted by fine-tuning the composition of GNRs and PNIPAM during the process of self-assembly to form nanoparticles with shell-core structure.

**Figure 2:**
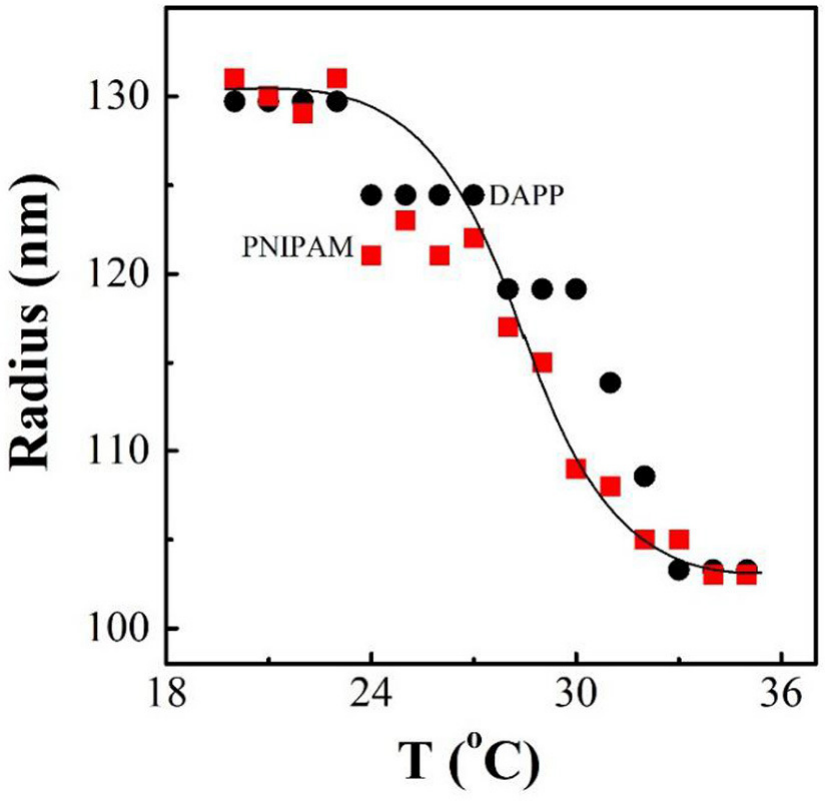
The temperature dependence of radius for PNIPAM and DAPP micelles in aqueous solutions at same concentration as tested by the dynamic laser light scattering (DLS).

**Figure 3:**
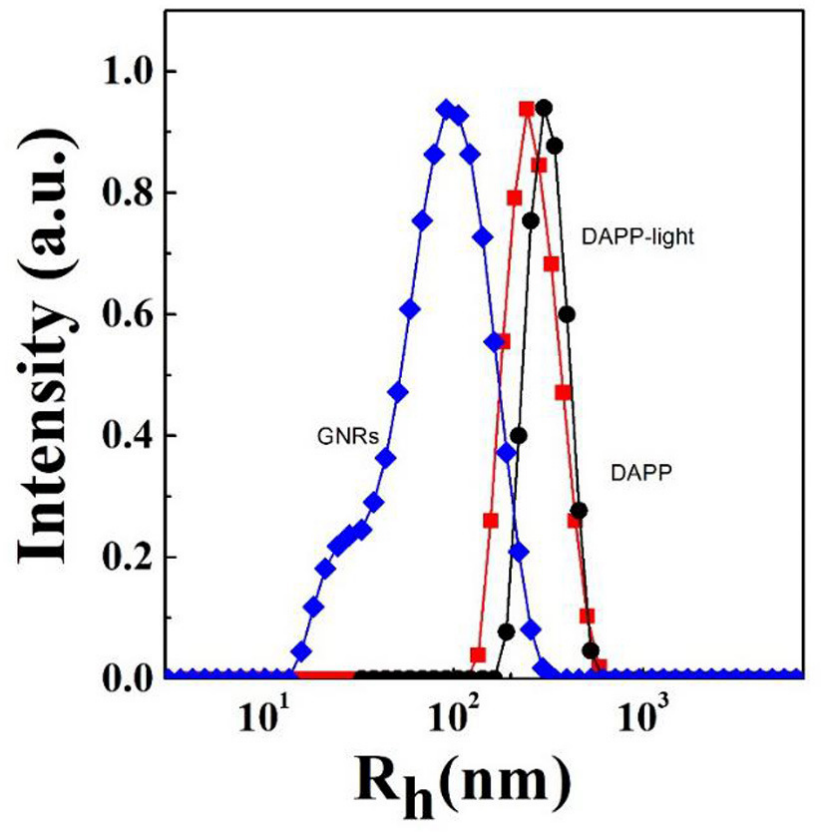
The hydrodynamic radius (R_h_) and size distribution of GNRs, DAPP with NIR-light, and DAPP as measured by DLS.

It is well-known that the lower critical solution temperature (LCST) of PNIPAM is about 32°C, but it can be modified by different methods of polymerization. In this study, the thermosensitive homopolymer PNIPAM was physically entrapped inside the core of the micelles. The volume phase transition temperature (VPTT) of the DAPP sample was similar to the LCST of the PNIPAM, indicating that the polymer synthesized after loading GNRs and DOX still exhibited a good temperature response (Figure 2). The size and size distribution of GNRs, DAPP, and DAPP with NIR-light were evaluated by DLS as shown in Figure 3. It was clear that the GNRs were entrapped into the micelles, and the size was shifted to the right with a hydrodynamic radius in the range of 200 to 300 nm. However, as the DAPP was irradiated by the NIR light, and the size of the DAPP became smaller. The size changes indicated that photothermal transition and PNIPAM shrinking had occurred, and ultimately indicated the successful composition and structure construction. This photothermal transformation of GNRs can be effectively used to promote the shrinking of PNIPAM inside of DAPP and to further control the DOX release inside the tumor lesion.

**Figure 4:**
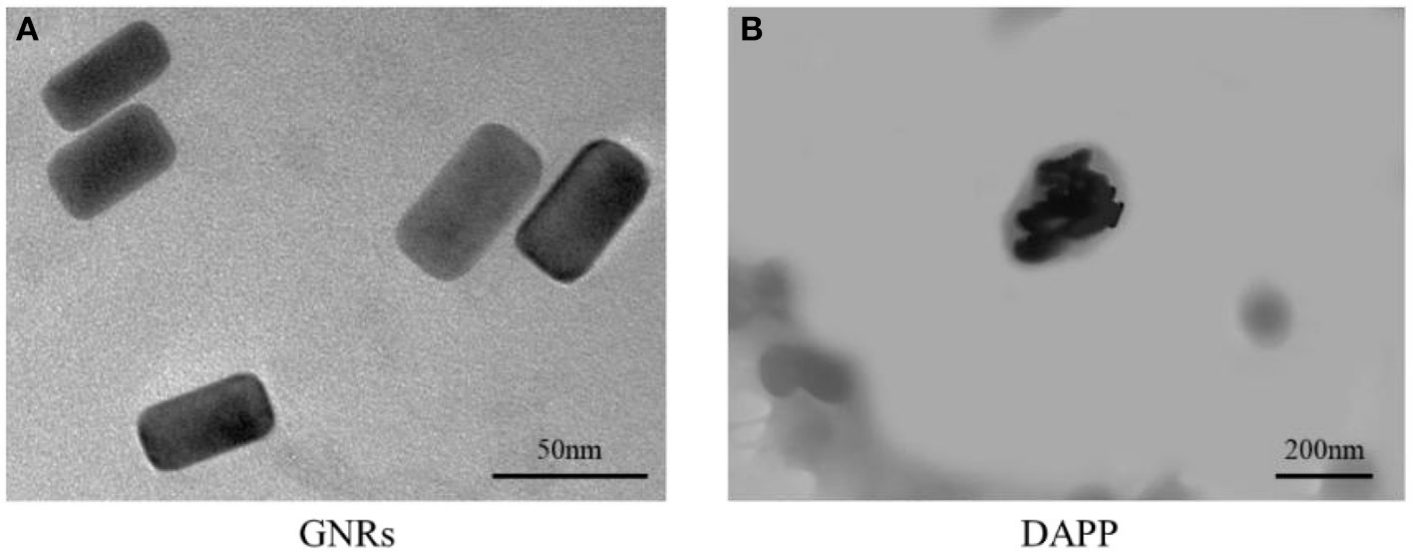
TEM image of GNRs **(A)** and DAPP micelles **(B)**.

**Figure 5:**
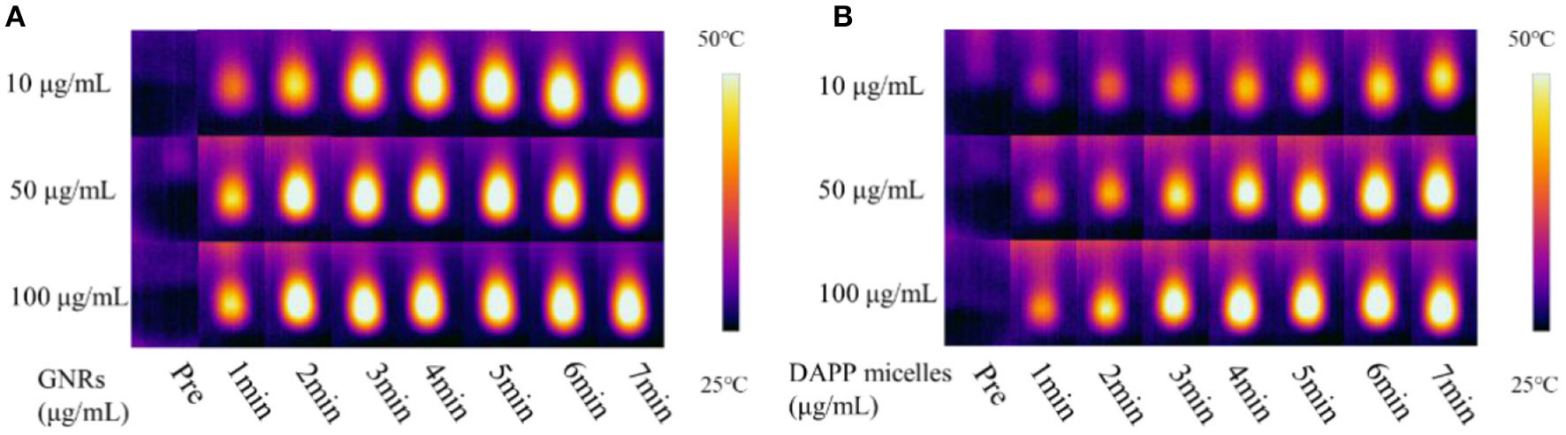
The photothermal transition efficiency of GNRs **(A)** and DAPP **(B)**.

The TEM morphology of GNRs and DAPP are shown in Figures 4A,B. The particle sizes of GNRs and DAPP obtained from TEM were similar to that of DLS. The photothermal conductivity efficiencies of GNRs and DAPP at the same concentration but at different times are shown in Figure 5. Both the temperature of the GNRs solution (left panel) and DAPP solution (right panel) increased with the time and concentration exposed to light. The excellent photothermal conversion efficiency could be used to regulate the PNIPAM phase transition and drug release inside tumors. Overall, the preceding evidence indicated that the temperature and light-sensitive dual-functional DAPP was successfully constructed.

### Serum Stability and Release Profile of DAPP

**Figure 6:**
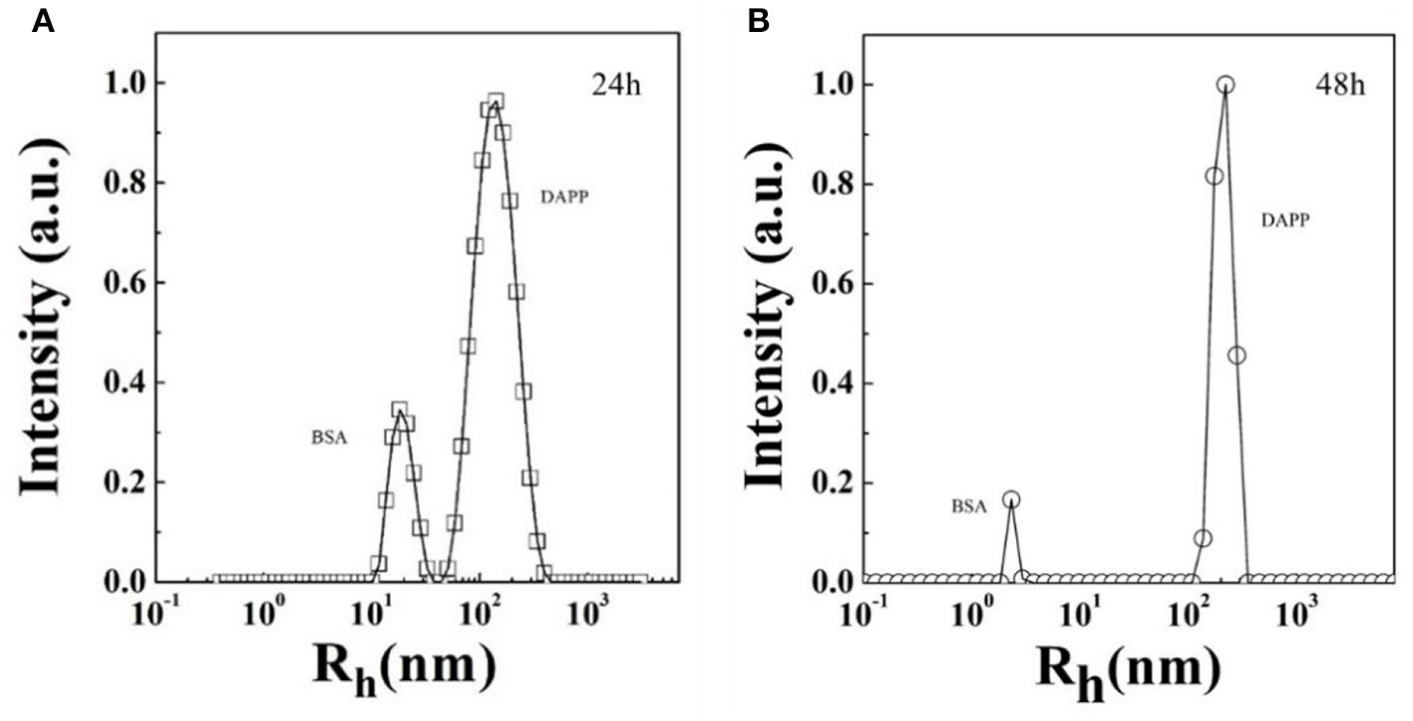
**(A,B)** The serum stability of DAPP micelles as tested by the blend of BSA with DAPP at 24 h and 48 h.

Serum stability and successful intratumoral drug release are two key factors strongly affecting drug accumulation. Here, the serum stability of DAPP was tested by checking the size and size distribution of DAPP and its blending with serum protein BSA, which was carefully checked by the DLS as shown in Figure 6. It is clear that there were two well-separated peaks with sizes about 10 and 200 nm. The small peak was similar to that of BSA, which is in the range of 1–10 nm. Conversely, the larger peak was about 200–300 nm. As mentioned above, the size of the DAPP obtained both in the DLS and on TEM analyses was about 200 nm. Thus, the larger peak in Figure 6 reflected the size of the DAPP. In DLS, the peaks can be utilized to analyze the interaction between particles. Theoretically, the size of BSA and DAPP will merge together if there is an interaction between BSA and DAPP, which includes the absorption of BSA onto DAPP, the charge neutralization, and chain entanglement. The size will increase markedly with a broad distribution. Both Figures 6A,B shows that the two peaks were well-defined at different detecting time 24 and 48 h, which indicates that the DAPP holds high serum stability.

**Figure 7:**
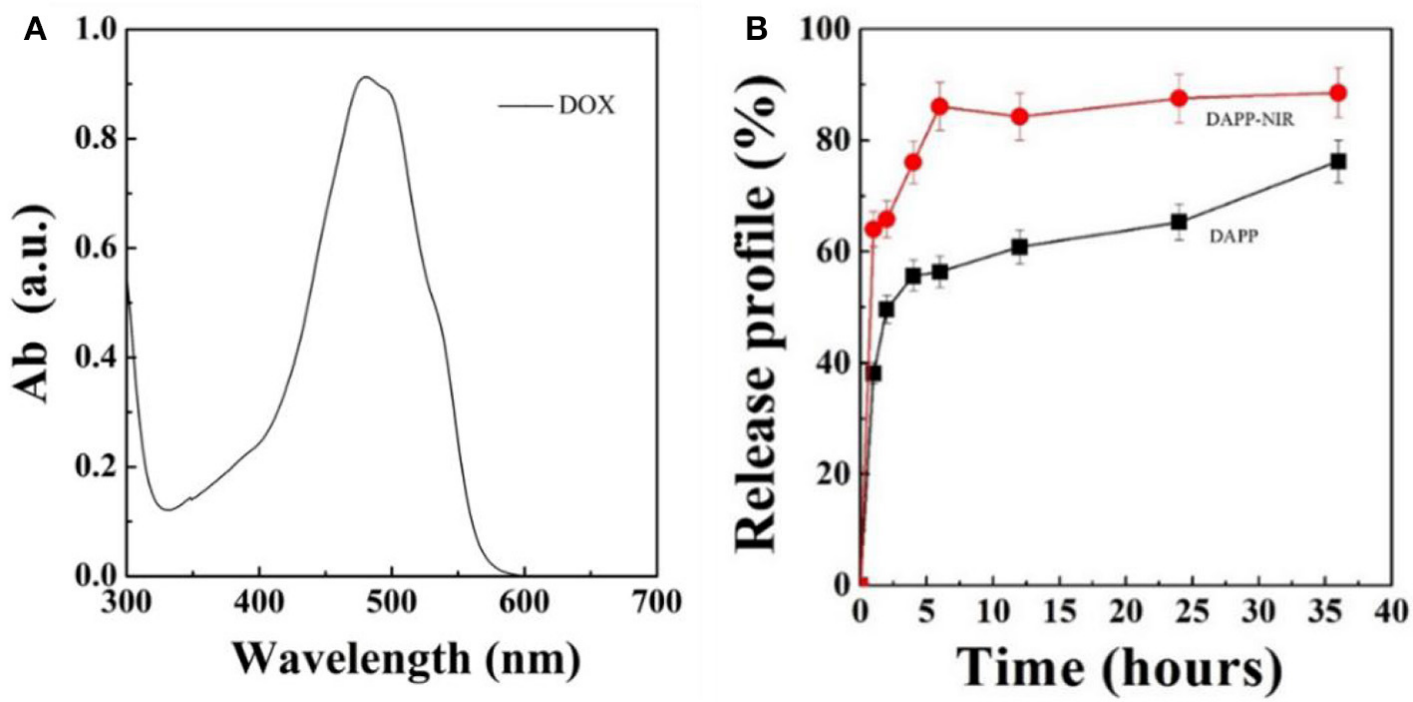
Optical absorption curve of DOX **(A)** and *in-vitro* DOX releasing profile of DAPP and DAPP with NIR-light at 37°C **(B)**.

Figure 7 shows the DOX release profile. As mentioned above, intratumoral drug-release plays an important role in the therapeutic efficiency when the DAPP accumulates in the tumor. In this study, we used NIR-light as a sensor to trigger the photothermal transition through the GNRs. The collapse of the PNIPAM was triggered by the heat generated and was followed by an increase in DOX release. The *in-vitro* release profile of DOX from DAPP was then regulated intratumorally. Figure 7 shows that the maximum absorption wavelength of DOX is 480 nm and the DOX concentration increased after NIR-light because the drug release rate of the DAPP with NIR-light was significantly higher than that of DAPP without NIR. Compared with the DAPP (non-NIR), about 90% of DOX was released within 10 h, which indicated that GNRs and PNIPAN loaded in the core of the DAPP had performed as expected and was suitable for promoting intratumoral drug accumulation.

### *In-vitro* Cellular Internalization and Cytotoxicity

**Figure 8:**
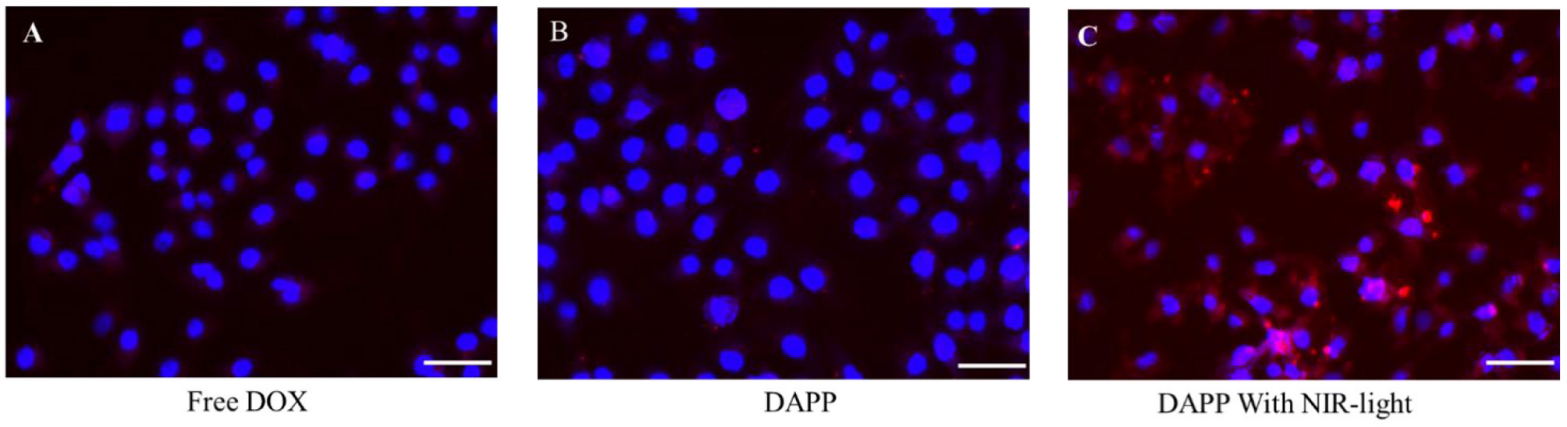
The B16F10 cellular uptake evaluation. Free DOX **(A)**, DAPP **(B)**, and DAPP with NIR-light **(C)**, the scale bar:100 μm.

After intratumoral accumulation and drug release, tumor cell capture and cytotoxicity are the next most important issues for tumor inhibition. Herein, the cellular internalization was monitored by inverted fluorescence microscopy as shown in Figure 8. The blue fluorescence indicates the nucleus stained by DAPI and red fluorescence is from DOX. The image clearly shows that the cellular uptake of DAPP with NIR-light (Figure 8C) was much higher than of free-DOX (Figure 8A) and DAPP without the NIR-light trigger (Figure 8B). Therefore, it can be easily concluded that DAPP could deliver the drug to tumor cells. Moreover, the drug could be stimulated to be released in tumor cells by exposure to NIR-light and produced a high intratumoral accumulation of DOX.

**Figure 9:**
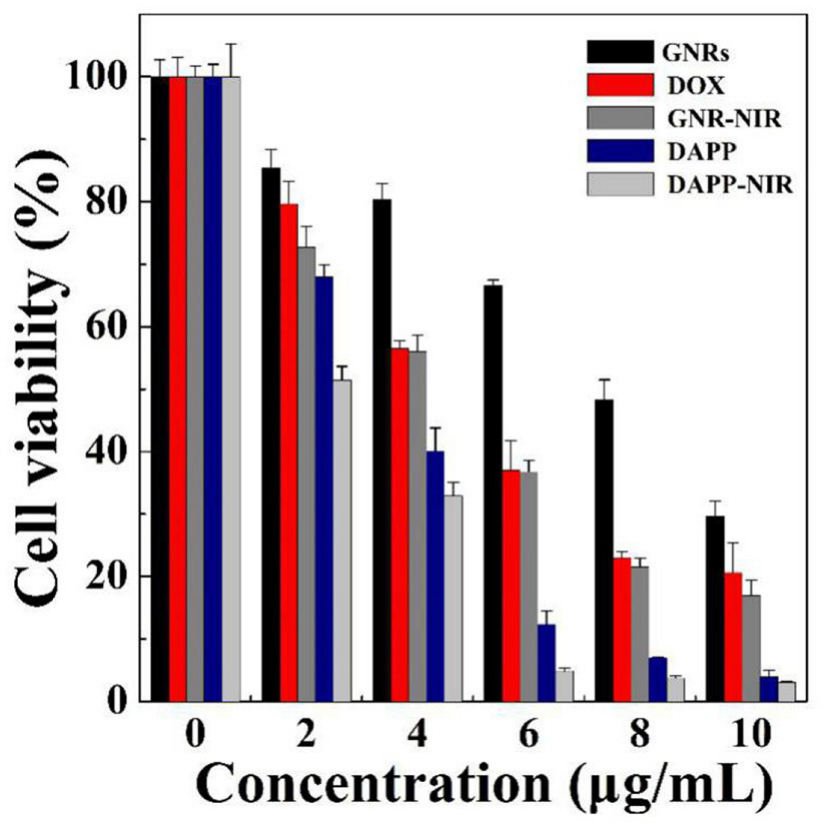
*In-vitro* evaluation of the cytotoxicity of DAPP against B16F10 cells.

The *in-vitro* cytotoxicity of GNRs, DOX, and DAPP against B16F10 melanoma cells was further evaluated by the CCK-8 assay as shown in Figure 9. The cytotoxicity assay was conducted under NIR-light. At the same concentrations and time points, the cytotoxicity of DAPP with NIR-light was much higher than that of free-DOX or free-GNR. The higher temperature generated by GNRs under the NIR-light could also kill cancer cells, which also resulted in higher cytotoxicity by GNRs. This indirectly suggested that GNRs had a good photothermal conversion effect. This cytotoxicity results were similar to our previous studies, in which the intracellular mechanism of GNRs to selectively kill cancer cells under the conditions of NIR-light was systemically investigated (Zhang et al., 2017). Therefore, the cytotoxicity of the temperature/light dual-functional DAPP micelles correlated with the increased cellular uptake and enhanced intracellular drug release shown in Figure 8.

### *In-vivo* Biodistribution and Antitumor Effects of DAPP

**Figure 10:**
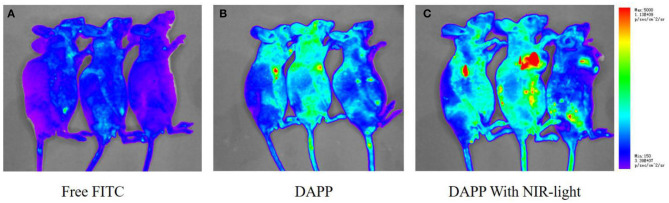
The *in-vivo* biodistribution evaluation. The biodistribution of free FITC **(A)**, DAPP **(B)**, and DAPP with NIR-light **(C)**.

The *in-vivo* distribution of DAPP micelles monitored by the florescent marker FITC in the melanoma tumor-bearing nude mice is shown in Figure 10. From the living animal images, the red fluorescent area of DAPP detected for the NIR light-treated group was obviously higher than that of DAPP without NIR-light treatment and FITC-free groups, which indicated that the DAPP could significantly promote drug accumulation at the tumor site. This enhanced tumor accumulation of FITC is partly due to the EPR effect, which is dominated by the PEG chain surrounding the DAPP. Meanwhile, the high fluorescent intensity of DAPP with NIR-light is mainly attributable to the GNRs photothermo transition, which promotes the release of on-site fluorescent molecule release at the same site as intratumor accumulation of DAPP. Therefore, the DAPP with NIR-light treated group promoted the concentration of DOX at the tumor sites, which was significantly improved by NIR-light. Noted here, the experiments about biodistribution and biosafety of DAPP were conducted while the data was not shown here, which was analyzed and used in our other work.

**Figure 11:**
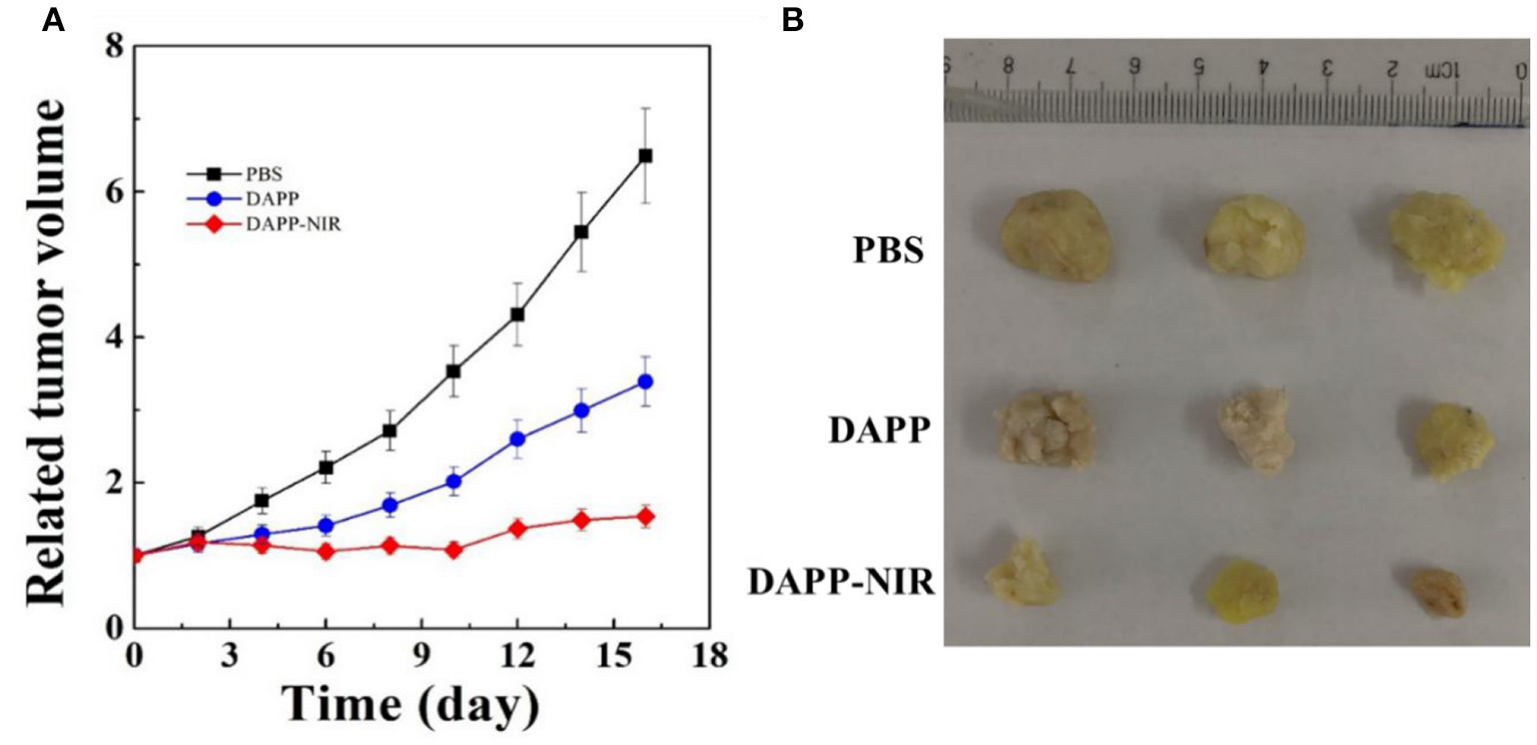
The *in-vivo* antitumor effect of PBS, DAPP, and DAPP-NIR **(A)** and the volume of solid tumor harvested from different groups **(B)**.

Figure 11 shows the *in-vivo* antitumor effects evaluated in melanoma tumor-bearing nude mice. When the tumor grew to around 100 mm^3^, the mice were divided into three treatment groups: PBS, DAPP, and DAPP with NIR-light. Compared with DAPP without NIR-light exposure, the relative tumor volume was much smaller. This clearly indicated that the intratumor drug release was successfully enhanced by the NIR exposure due to the photothermal transition followed by the thermo-regulated drug release. Figure 11 shows that the relative tumor volume of the PBS-treated group, that is, the control group, was the largest, which indicated that the EPR effect of the nano-sized DAPP was promoted by both the size and the PEG chain (Figure 10). Consequently, the tumor volume of the control group was about 6-times larger than that of DAPP-NIR light-treated group, which is attributed to both the EPR effect and the photothermo transition of GNRs inside of DAPP of the treatment groups. It may be concluded that the concentration of DOX release as delivered by the DAPP micelles at the tumor site was regulated by physical stimuli represented by the NIR-light. In addition, as shown in Figure 11B, the therapeutic effects were also clearly reflected in the tumor tissue harvested from the corresponding groups. Moreover, both our's and other's previous works confirmed that GNRs exposed specific toxicity to tumor cells, while there was little cytotoxicity to non-tumor cells (Wang et al., 2011; Zhang et al., 2017).

**Figure 12:**
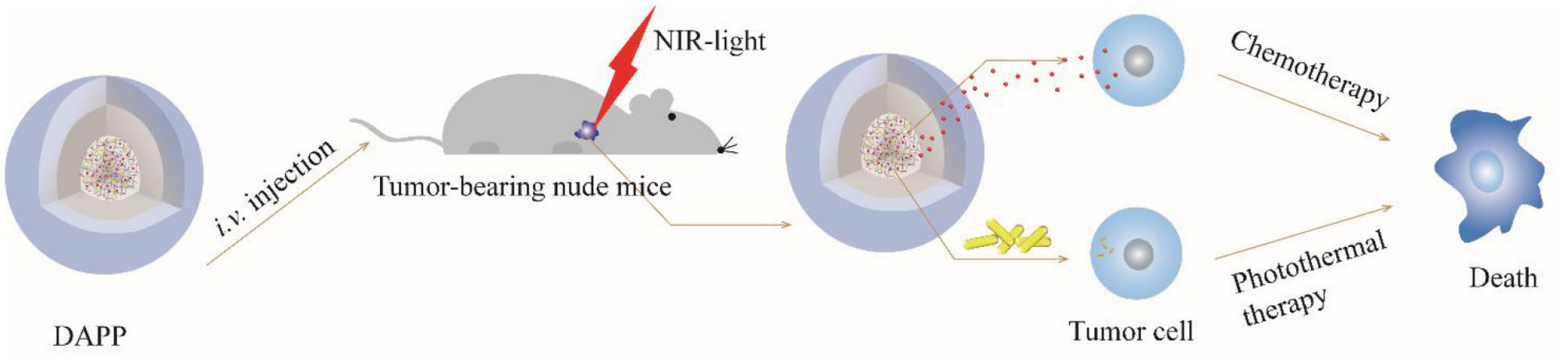
This scheme illustrates the *in-vitro/in-vivo* mechanism of the DAPP.

Taken together, the overall mechanism of DAPP activity is illustrated in Figure 12. DOX and GNRs are combined with PNIPAM, and then loaded in photo- and thermo-sensitive micelles, which act as dual-function DAPP. These bifunctional nanomicelles were successfully prepared by fine-tuning physical properties as described in our previous study (Su et al., 2017). The particle size of the DAPP was about 220 nm and presented a spherical shape, and its construction was tailored to exhibit dual-functions, good biocompatibility, excellent photo and thermal sensitivity, and good serum stability. The release of DOX in DAPP micelles showed a greater dependency on the GNRs photothermal conversion effect and on the temperature responsiveness of PNIPAM, which strongly influenced its therapeutic activity against melanoma. The *in-vitro* cytotoxicity assay showed that the GNRs and the chemotherapeutic drug DOX exhibited a synergistic effect on the killing of B16F10 cells. The results of the *in-vivo* distribution and tumor inhibition assay showed that DAPP micelles were more highly enriched at tumor sites, which was partly due to the EPR effects of DAPP. In addition, the tumor inhibition effect of DAPP under NIR-light-treatment was even superior to that of other groups, because the DAPP micelles system can control PNIPAM shrinkage and lead to higher in-site tumor cellular drug release and thus promote a stronger chemotherapeutic effect.

## Conclusion

In summary, temperature and light-sensitive dual-functional DAPP micelles were successfully prepared by a fine-tuned physical-chemical self-assembly and exhibit a well-defined shell-core structure and excellent biocompatibility. First, the EPR effect of DAPP promoted the accumulation of micelles at the tumor site. Then, the photothermal effect of GNRs in the DAPP core further enhanced the drug release in tumor cells regulated by the PNIPAM collapse, which in turn was regulated by the NIR-light-induced photothermal transition, which triggered the release of a high concentration of DOX at the tumor site. In addition, the advantages of this dual functional DAPP included the sparing of normal tissue as compared with previous strategy by *in-vivo* tissue ablation. The experimental results showed that DAPP induced excellent antimelanoma effects both *in vitro* and *in vivo* under NIR-light-triggered drug release. Consequently, this study supports the use of the DAPP micelles construct as an effective nanodrug delivery system with high treatment potential against melanoma through a physical stimuli-responsive cargo release, and may represent a novel approach to the clinical therapy of melanoma.

## Data Availability Statement

The original contributions presented in the study are included in the article/supplementary materials, further inquiries can be directed to the corresponding author/s.

## Ethics Statement

The animal study was reviewed and approved by the Institutional Review Board of the Second Military Medical University.

## Author Contributions

All authors listed have made a substantial, direct and intellectual contribution to the work, and approved it for publication.

## Conflict of Interest

The authors declare that the research was conducted in the absence of any commercial or financial relationships that could be construed as a potential conflict of interest.
